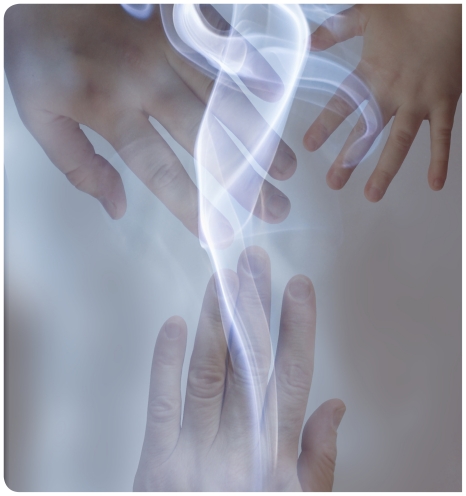# SMOKING AND SECONDHAND SMOKE: Global Estimate of SHS Burden

**DOI:** 10.1289/ehp.119-a66

**Published:** 2011-02

**Authors:** Naomi Lubick

**Affiliations:** **Naomi Lubick** is a freelance science writer based in Stockholm, Sweden, and Folsom, CA. She has written for *Environmental Science & Technology*, *Nature*, and *Earth*

Call it what you will—passive smoking, environmental tobacco smoke, or secondhand smoke (SHS)—worldwide, exposure to the emissions from smokers’ cigarettes caused the premature death of an estimated 603,000 people in 2004, according to a team of academic and World Health Organization (WHO) researchers.^1^ This first global assessment of the burden of SHS was led by Mattias Öberg of the Karolinska Institute and sponsored by the Swedish National Board of Health and Welfare and Bloomberg Philanthropies.

SHS was first confirmed in the mid-1980s to cause adverse health effects.^2^ The associated effects now include heart disease, lung cancer, worsening of asthma, sudden infant death syndrome, and more. But until now, data on deaths and disease among nonsmokers have not been compiled at the global scale.

“There have been estimates made before for specific countries on the impact of SHS,” says epidemiologist Jonathan Samet of the University of Southern California, who did not participate in the new study. “The new paper by Öberg and colleagues is important for putting together a global picture.”

The researchers searched the scientific literature, public health reports, and government databases for reliable data on smoking according to age and sex. Where needed data did not exist, they created models to extrapolate from well-studied regions to countries with low data availability. One main data source for exposure among children was the Global Youth Tobacco Survey,^3^ cosponsored by the U.S. Centers for Disease Control and Prevention in more than 120 countries. The school-based survey is administered annually to 13- to 15-year-olds to assess children’s use of tobacco products and exposure to SHS.

Öberg says the team took a conservative approach in its assessment. For example, the researchers chose not to include deaths or diseases without clear and strong evidence for a causal relationship to SHS exposure (stroke was one such disease). They excluded diseases that have been causally linked to SHS if strong and comparable international health statistics were unavailable (one such example was sudden infant death syndrome). The team also did not include premenopausal breast cancer, as the relationship between this disease and SHS remains controversial in the scientific community.^4^

“It’s difficult to find really good data [for some of these outcomes],” Öberg says. Moreover, he adds, it’s hard to relate some effects directly to SHS exposure. For instance, despite strong suggestive evidence of links between stroke and chronic obstructive pulmonary disease (COPD) and SHS, these links have not been confirmed by strong epidemiologic meta-analyses.^1^

Finding high-quality peer-reviewed SHS exposure data also has proved difficult; far more data are available on the number of active smokers, Öberg says. He adds that, although smokers themselves are likely affected by passive smoking, they were not included in the team’s main assessment. If they had been included, the estimated mortality rate would have increased by about 30%; ex-smokers were included, and without them, the total number of deaths would decrease by 17%.^1^

In the end the team estimated the global proportion of people exposed to SHS in various settings, as of 2004, at 40% of all children (defined as age 0–14 years), 33% of nonsmoking men, and 35% of nonsmoking women around the world. But those proportions varied by region according to smoking habits, rural versus urban populations, country regulations, and other factors. For example, in the region encompassing Belarus, Estonia, Hungary, Kazakhstan, Latvia, Lithuania, Republic of Moldova, the Russian Federation, and Ukraine, around two-thirds of nonsmokers in all age and sex groups were estimated to be exposed. In southern and northeast Africa, only 12% of children and even fewer men and women were estimated to be exposed.^1^

The burden of morbidity from SHS exposure, as measured by disability-adjusted life years (DALYs), also varied by region, with higher estimates for low-income countries in Southeast Asia and the eastern Mediterranean region compared with Europe. Asthma and ischemic heart disease accounted for the most disease among adults, and lower respiratory infections were the most common outcome among children.^1^

Most striking, children under age 5 years bore the brunt of respiratory infections in poorer countries, where malnutrition or inadequate health care also may lead to higher disease and mortality rates in children with other health problems that are exacerbated by SHS exposure. The team calculated that children overall experienced an estimated 61% of the disease burden from SHS.^1^

“Children remain exposed in the home,” even in countries with legislation that removes smoking from public places, says Heather Wipfli, a University of Southern California policy expert who, with Samet, coauthored a comment in *The Lancet*^5^ on the new research. But Wipfli considers SHS exposure largely a women’s issue: only about 10% of women in the world smoke, she explains, but of the 603,000 SHS-related deaths of nonsmokers estimated in 2004, 47% were among women (compared with 26% among men and 28% among children).^1^

No matter what assumptions you use, the impact [of SHS] on children and adults is still of great public health significance.

**–Bart Ostro**

**Centre for Research in Environmental Epidemiology**

In one bit of good news, Wipfli says China’s percentage of women smokers has remained low, despite concerns among the public health community that Chinese women would be a market targeted by the tobacco industry—traditionally, women there have not smoked, something that growing wealth and commercialism might have changed. Still, Europe and Asia, and particularly lower-income countries in those regions and countries where almost all parents smoke at home, have extremely high SHS exposures for nonsmokers in general, Wipfli says. She and Samet urge the full implementation of the various components of the WHO Framework Convention on Tobacco Control (an international treaty that works on both supply and demand for tobacco) and associated policy and educational programs.

The WHO will report next year on how many nations have passed bans on smoking in public spaces, including work sites and restaurants, says Armando Peruga, a program manager with the WHO Tobacco Free Initiative and coauthor of the *Lancet * report.^1^ Peruga says the team needs to do additional work to refine their estimates and gather more data for individual countries, particularly those lacking complete reporting data on smoking; he hopes the team may have these calculations completed in a year or so.

Meanwhile, the new study is “an impressive effort at producing an estimate of the global effects of secondhand smoke,” says Bart Ostro, a research scientist at the Centre for Research in Environmental Epidemiology (CREAL) in Barcelona, on temporary leave from the California Environmental Protection Agency. Ostro comments that the epidemiologic method the team used to reach its conclusions is well established and that they “utilized a lot of studies that have been heavily peer reviewed in the past,” including the U.S. Surgeon General’s seminal 2006 report and a similar 2005 report from the California Environmental Protection Agency.

The researchers’ sensitivity analysis “shows that no matter what assumptions you use, the impact on children and adults is still of great public health significance,” Ostro adds.

Despite remaining gaps in the data, the estimate is “a policy-relevant number and one that should motivate action,” says Samet. “These exercises are intended to provide general guidance and an understanding of the magnitude of the disease burden . . . and how much could be avoided by preventive strategies.”

## Figures and Tables

**Figure:**